# Pre-symptomatic modified phytohormone profile is associated with lower phytoplasma titres in an Arabidopsis *seor1ko* line

**DOI:** 10.1038/s41598-020-71660-0

**Published:** 2020-09-08

**Authors:** Chiara Bernardini, Laura Pagliari, Valeria De Rosa, Marilia Almeida-Trapp, Simonetta Santi, Marta Martini, Sara Buoso, Alberto Loschi, Nazia Loi, Fiorella Chiesa, Axel Mithöfer, Aart J. E. van Bel, Rita Musetti

**Affiliations:** 1 Department of Agricultural, Food, Environmental and Animal Sciences, University of Udine, via delle Scienze, 206, 33100 Udine, Italy; 2 Department of Bioorganic Chemistry, Max Planck Institute for Chemical Ecology, Hans-Knöll-Straße 8, 07745 Jena, Germany; 3 Research Group Plant Defense Physiology, Max Planck Institute for Chemical Ecology, Hans-Knöll-Straße 8, 07745 Jena, Germany; 4 Institute of Phytopathology, Justus-Liebig University, Heinrich-Buff-Ring 26–32, 35392 Giessen, Germany

**Keywords:** Biotic, Microbe

## Abstract

The proteins AtSEOR1 and AtSEOR2 occur as conjugates in the form of filaments in sieve elements of *Arabidopsis thaliana*. A reduced phytoplasma titre found in infected defective-mutant *Atseor1ko* plants in previous work raised the speculation that non-conjugated SEOR2 is involved in the phytohormone-mediated suppression of Chrysanthemum Yellows (CY)-phytoplasma infection transmitted by *Euscelidius variegatus* (Ev). This early and long-lasting SEOR2 impact was revealed in *Atseor1ko* plants by the lack of detectable phytoplasmas at an early stage of infection (symptomless plants) and a lower phytoplasma titre at a later stage (fully symptomatic plants). The high insect survival rate on *Atseor1ko* line and the proof of phytoplasma infection at the end of the acquisition access period confirmed the high transmission efficiency of CY-phytoplasma by the vectors. Transmission electron microscopy analysis ruled out a direct role of SE filament proteins in physical phytoplasma containment. Time-correlated HPLC–MS/MS-based phytohormone analyses revealed increased jasmonate levels in midribs of *Atseor1ko* plants at an early stage of infection and appreciably enhanced levels of indole acetic acid and abscisic acid at the early and late stages. Effects of Ev-probing on phytohormone levels was not found. The results suggest that SEOR2 interferes with phytohormonal pathways in Arabidopsis midrib tissues in order to establish early defensive responses to phytoplasma infection.

## Introduction

Phytoplasmas affect hundreds of agronomically important plant species worldwide, including ornamentals, vegetables and fruit trees^1^, causing profound alterations in plant cytology and physiology, by modulation of transcript and protein profiles^2,3^ and changes in the hormonal balance (for a review see^4^).

Phytoplasmas are prokaryotic plant pathogens belonging to the class *Mollicutes*. In plant hosts, phytoplasmas are restricted to the sieve elements (SEs)^5^, which are responsible for the translocation of nutrients and a broad spectrum of signals. Phloem-feeding insects act as vector hosts that contaminate healthy plants with phytoplasmas ingested during previous probing of infected plants^6^.

In Arabidopsis, two non-redundant Sieve-Element Occlusion Related (SEOR) genes, *AtSEOR1* (At3g01680) and *AtSEOR2* (At3g01670)^7^, were reported to be necessary for the formation of SE protein filaments through a heteromeric assemblage of the two SEOR proteins^8^. Studying the role of SEOR proteins following Chrysanthemum Yellows (CY)-phytoplasma infection in *Arabidopsis thaliana*, Pagliari et al.^9^ noted that the *Atseor1ko* mutant line hosted a considerably lower number of phytoplasmas, even though the phloem flow (and thus the pathogen spread capability) is not affected^9^. This observation led to the hypothesis that an unknown SEOR2-associated mechanism assists the plant to combat the pathogen. It matches the idea that AtSEOR2 proteins, which are not conjugated with SEOR1, as is the case in *Atseor1 ko* plants, may be involved in plant immune responses^10,11^ or in phytohormone-mediated signalling pathways^12^.

Phytohormones are key mediators in plant adaptation to environmental changes^13^ and, hence, are also engaged in responses to beneficial^14^ and pathogenic microorganisms^15^. In general, phytohormones sustain a dynamic network to optimize plant-responsive processes^16^. A number of studies addressed phytohormone levels in phytoplasma-infected plants (for a review see^4^). In accordance with this information, increased salicylic acid (SA) and abscisic acid (ABA) levels and a decreased indole-3-acetic acid (IAA) content are to be expected as responses to phytoplasma infection, while the picture for other phytohormones is unclear^4^. Simultaneous analyses of more than two phytohormones during phytoplasma infections are scarce^17, 18^ and give rise to contrasting conclusions. The conflicting results may be due to the use of diverse pathosystems (model plants^19^; field-grown woody plants^20^), different sampling methods (whole leaves^21^; leaf midribs^22^; seeds^23^; or phloem sap^24^), and dissimilar techniques (phytohormone quantification by HPLC analysis^21^; gene expression analyses^20,25^). All in all, the phytohormonal response(s) to phytoplasma infection remain(s) largely unclear thus far.

We investigated whether phytohormonal production is associated with the presence of free SEOR2 and, if so, which phytohormone levels at which stage are affected by phytoplasma infection. An attempt was made to localize the phytohormone production. To this end, phytohormone levels were monitored both in the midribs and laminar tissues of wild-type, *Atseor1ko* and *Atseor2ko* Arabidopsis lines at the beginning and at the end of the phytoplasma infection. As phytohormone synthesis could also have been activated by the leafhopper vector *Euscelidius variegatus*^26^, Arabidopsis plants not infested by leafhoppers and plants infested by leafhoppers free from phytoplasmas were analysed in parallel. Furthermore, phytoplasma titre was quantified at the same infection stages and leafhopper survival rates were evaluated to exclude differences in insect-mediated transmission efficiency in the three Arabidopsis lines. As a structural element, transmission electron microscopy (TEM) analyses were performed to determine possible structural modifications brought about by phytoplasma infection.

## Results

### Phytoplasma transmission by *Euscelidius variegatus*

In this study, three treatment groups of the three Arabidopsis plant lines were investigated: (1) plants (no-Ev) not infested by *E. variegatus*, (2) plants (H-Ev) infested by *E. variegatus* from a healthy colony that never fed on phytoplasma-infected plants*,* and (3) plants (CY-Ev) infested by CY-infected *E. variegatus*. For each analysis plants were tested at two stages, i.e. 5 (T1) and 20 days (T2) after the end of the inoculation access period (IAP), which are referred to as the “early” and “late” stage of infection, respectively.

Three individuals of healthy *E. variegatus* or CY-infected *E. variegatus* were placed on wild-type, *Atseorko1* and *Atseor2ko* Arabidopsis plants. The insects were manually removed after 7 days (i.e*.* at the end of IAP) and the number of living individuals was counted. The survival rates of healthy *E. variegatus*, which ranged between 59 and 79% (Fig. S1), showed no statistically significant differences between the respective Arabidopsis lines. Yet there was a tendency for the healthy individuals to survive slightly better on the *Atseorko* mutants (Fig. S1). This tendency became statistically significant (Fig. S1) for CY-infected-*E. variegatus*, which suffered from decreased fitness on wild-type plants as compared to the mutants (with a lower survival rate, ranging from 19.5 to 38.9%).

Healthy and CY-infected *E. variegatus* were then pooled (as reported below) and processed for phytoplasma detection. PCR analysis, with a 1,250 bp amplicon as a final product, confirmed the presence of phytoplasmas in all pools from 3 insects having fed on CY-infected chrysanthemum. The success of the infection in CY-Ev plants was further confirmed by symptom development in each Arabidopsis line under investigation (100% plants were positive to CY phytoplasma following inoculation with vectors having fed on CY-infected chrysanthemum, with a transmission rate (p) of 1^27^.

**Figure 1:**
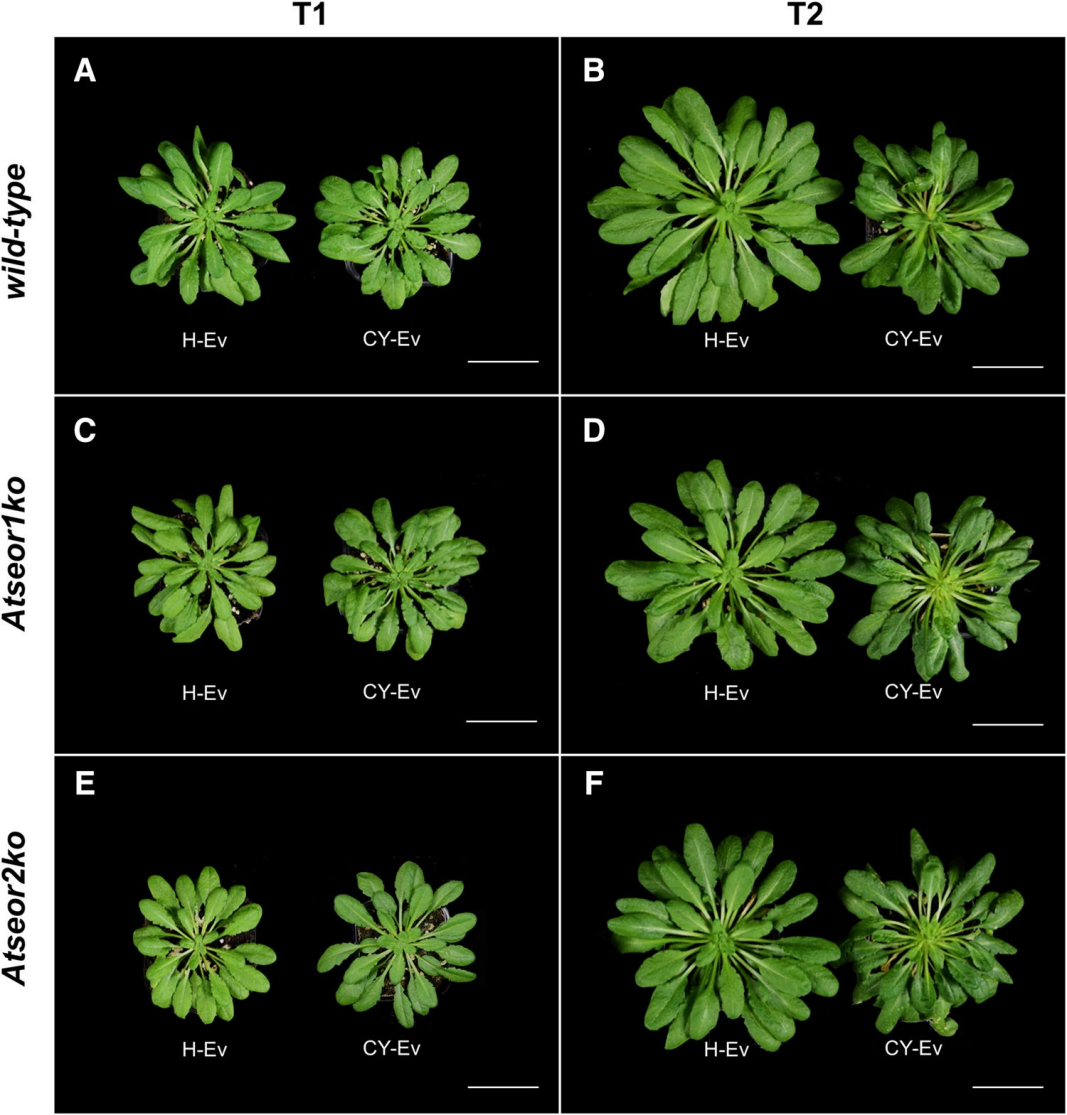
Phenotypes of *Arabidopsis thaliana* lines infested by healthy leafhoppers (H-Ev) or infested by CY-infected leafhoppers (CY-Ev) at the early (5 days after IAP, T1), and late stage (20 days after IAP, T2) of infection. The various conditions are indicated as follows: (**A**) and (**B**) wild-type, (**C**) and (**D**) *Atseor1ko* and **E** and **F** *Atseor2ko*, infested by healthy leafhoppers (H-Ev) or infested by CY-infected leafhoppers (CY-Ev). At an early infection stage (**A**,**C**,**E**) infected and healthy plants looked similar, symptoms manifested at the late (T2) stage (**B**,**D**,**F**), when chlorosis and leaf roll appeared. At both stages of infection, no differences between wild-type and the mutant lines were evident. Bars correspond to 5 cm.

Five days after the IAP, no symptoms were visible in the lines exposed to the CY-infected *E. variegatus* (Fig. 1A,C,E). Initial symptoms (leaf chlorosis and petiole elongation) emerged starting from the 14th day after IAP and characteristic CY symptoms became fully discernible 20 days after IAP (Fig. 1B,D,F). In fact, at this time point, all infected plant lines showed reduced growth and shorter, yellowish leaves, with a thick main vein. Chlorosis progressed from the youngest leaves towards the others. The appearance of the symptoms in wild-type and mutant lines was similar at this stage of infection (Fig. 1B,D,F).

### Phytoplasma quantification in Arabidopsis lines

To quantify the phytoplasma inside the CY-Ev Arabidopsis lines, qPCR was carried out using genomic DNA extracted from 12 plants for each infected Arabidopsis line.

**Figure 2:**
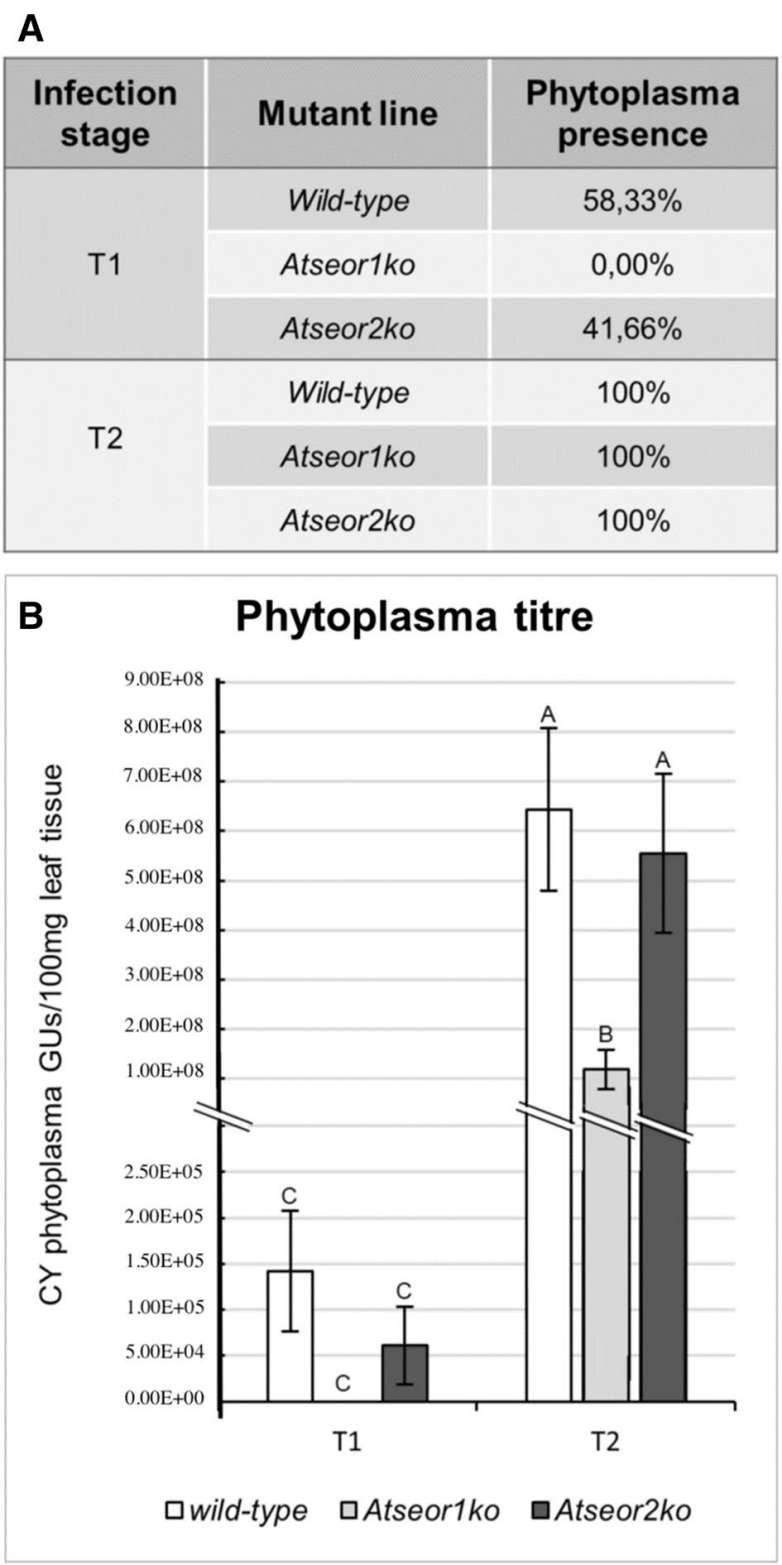
The CY phytoplasma titre and the percentage of infected plants at the early (5 days after IAP, T1) and late (20 days after IAP, T2) stage of infection. The percentage of infection (**A**) expresses the number of positive plants detected by real-time PCR out a total of 12 plants. Phytoplasma titre (**B**) is expressed as the number of CY phytoplasma genome units (GUs) per 100 mg of leaf sample to normalize the data. Different letters indicate different means according to the Holm-Sidak post hoc test, *P* < 0.05. Error bars indicate Standard Error of the Mean of 12 biological replicates for each condition.

At the early stage of infection (i.e. 5 days after IAP), none of the *Atseor1ko* plants tested positive for the presence of phytoplasma, while 58% of wild-type plants and 42% of *Atseor2ko* line did so (Fig. 2A). At this time-point, the phytoplasma titre was lower in comparison to that found at the late stage of infection, and did not significantly differ among the Arabidopsis lines (Fig. 2B). The highest cycle quantification (Cq) value to detect phytoplasma DNA in 100 mg of plant tissue was 32.83 for wild-type, corresponding to 7.76E+03 genome units (GUs), 34.30 for *Atseor2ko*, corresponding to 2.73E+03 GUs, while none of *Atseor1ko* plants resulted positive for phytoplasma presence. At the late stage of infection (i.e. 20 days after IAP), 100% of the plants treated with CY-Ev tested positive for phytoplasmas (Fig. 2A), but the phytoplasma titre in the *Atseor1ko* line was significantly lower than in *Atseor2ko* or wild-type plants (Fig. 2B and^9^). The highest Cq value to detect phytoplasma DNA in 100 mg of plant tissue was 18.38 for wild-type, corresponding to 2.05E+08 GUs, 18.07 for *Atseor2ko*, corresponding to 2.70E+08 GUs, 20.17 for *Atseor1ko* corresponding to 6.62E+08 GUs.

### Ultrastructural modifications in midrib phloem at the early and late stage of infection

**Figure 3:**
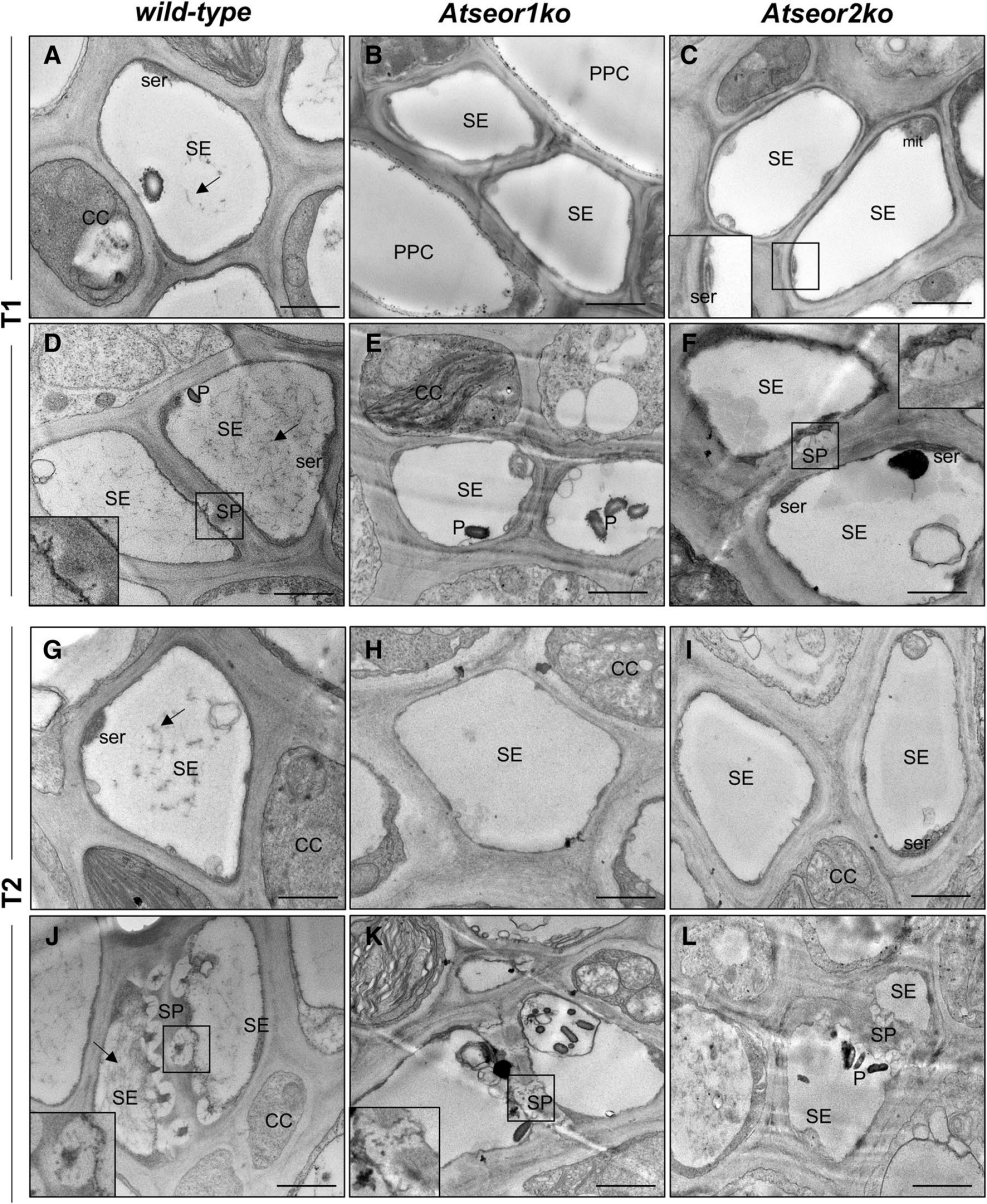
Ultrastructure of sieve elements in the three Arabidopsis lines not submitted to *Euscelidius variegatus* infestation (no-Ev plants). Phloem cross-sections were examined by TEM at T1 and T2s [i.e. respectively 5 (**A**–**F**) and 20 (**G**–**L**) days after the end of IAP] with the focus on structures in the SE lumen (**A**–**C**, **G**–**I**) and at the SPs (**D**–**F**, **J**–**L**). The wild-type line (**A**,**D**,**G**,**J**) contained SE protein filaments (black arrows). Both mutant lines, *Atseor1 ko* (**B**,**E**,**H**,**K**) and *Atseor2ko* (**C**,**F**,**I**,**L**), contained no protein filaments in either the SE lumen or in the SPs. *CC* companion cell, *mit* mitochondrion, *PPC* phloem parenchyma cell, *P* plastids, *SE* sieve element, *ser* sieve element reticulum, *SP* sieve plate. Bars correspond to 1 μm.

Both *AtSEOR1* and *AtSEOR2*^8,28^ are regarded as being necessary for the formation of SE protein filaments in Arabidopsis. As expected^8^, the SEs in the no-Ev wild-type line contained protein filaments, scattered throughout the SE lumen (Fig. 3A,G) and accumulated in the proximity of the sieve plates (Fig. 3D,J). On the contrary, the two no-Ev mutants, which are unable to form the respective SEOR partner proteins, showed no filaments in either the lumen (Fig. 3B,C,H,I) or near the sieve plates (Fig. 3E,F,K,L).

**Figure 4:**
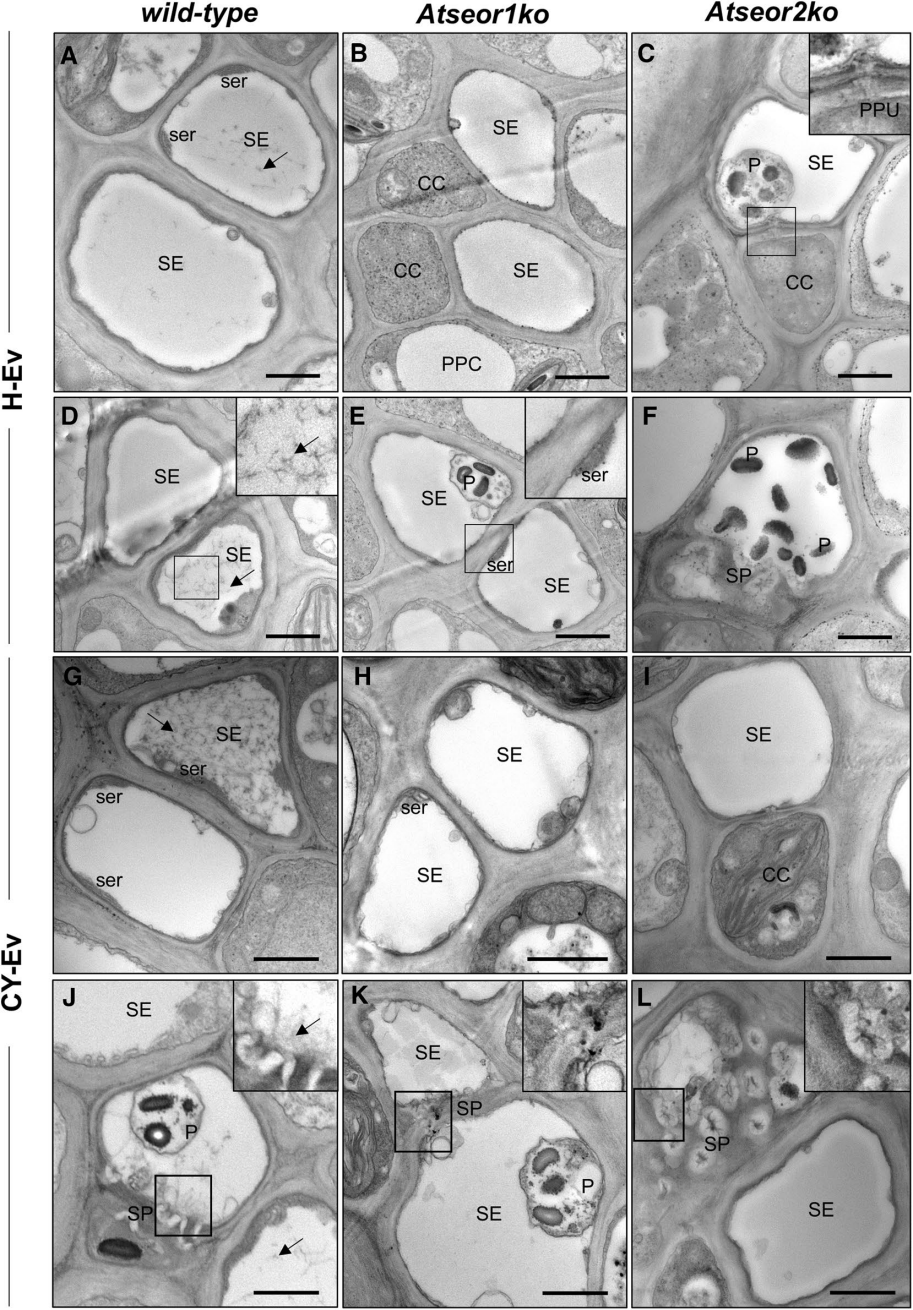
Ultrastructure of the sieve elements in three Arabidopsis lines at the early stage (5 days after IAP) of CY phytoplasma infection. Phloem cross-sections were examined by TEM in Arabidopsis lines infested by healthy leafhoppers (H-Ev, **A**–**F**) or infested by CY-infected leafhoppers (CY-Ev, **G**–**L**) with the focus on structures in the SE lumen (**A**–**C**, **G**–**I**) and at the SPs (**D**–**F**, **J**–**L**). The H-Ev (**A**,**D**) and CY-Ev (**G**,**J**) wild-type line contained SE protein filaments (black arrows). Mutant lines, both H-Ev (**B**,**C**,**E**,**F**) and CY-Ev (**H**,**I**,**K**,**L**), contained no protein filaments in either the SE lumen or in the SPs. In each Arabidopsis line, phytoplasmas were not detected. *CC* companion cell, *PPC* phloem parenchyma cell, *P* plastids, *PPU* pore-plasmodesma unit, *SE* sieve element, *ser* sieve element reticulum, *SP* sieve plate. Bars correspond to 1 μm.

In order to examine leafhopper- or phytoplasma-induced ultrastructural modifications in SEs, 6 H-Ev and 6 CY-Ev plants of each line were sampled for TEM analysis and compared with the corresponding no-Ev plants at 5 and 20 days after IAP. Five days after IAP, the H-Ev plants (Fig. 4A–F) did not show ultrastructural changes as compared to the no-Ev plants (Fig. 3). The SEs in the wild-type Arabidopsis showed protein filaments both in SE lumen (Fig. 4A) and near the sieve plates (Fig. 4D). As in the no-Ev plants (Fig. 3), the H-Ev mutant plants did not show filaments in the lumen of SEs (Fig. 4B,C) or near the sieve plates (Fig. 4E,F).

At the early stage (5 days after IAP), the CY-Ev wild-type plants (Fig. 4G,J) did not differ from their controls (Fig. 4A,D), in that protein filaments had accumulated in SEs (Fig. 4G,J). At this stage, *Atseor1ko* and *Atseor2ko* CY-Ev plants did not show any ultrastructural alterations as well, because SE filaments were not detected (Fig. 4H,I,K,L) just as in their controls (Fig. 4B,C,E,F). At this time-point, phytoplasmas were not found in TEM pictures in any of the CY-Ev Arabidopsis lines (Fig. 4G–L).

**Figure 5:**
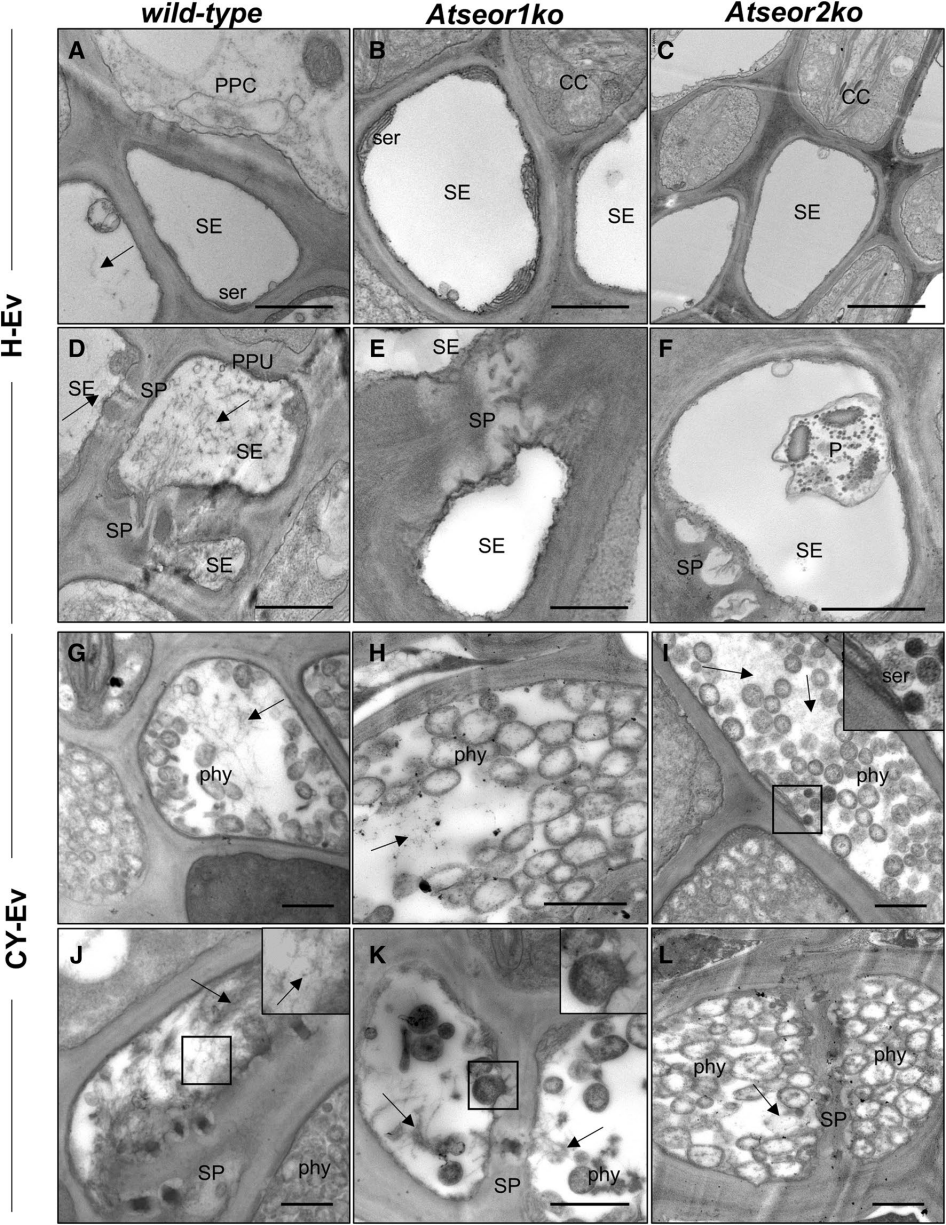
Ultrastructural modifications in three Arabidopsis lines at the late stage of infection. Phloem cross-sections of Arabidopsis lines infested by healthy leafhoppers (H-Ev, **A**–**F**) or infested by CY-infected leafhoppers (CY-Ev, **G**–**L**) were examined by TEM. SEs of H-Ev wild-type plants (**A**,**D**) contain protein filaments (black arrows) dispersed in the lumen (**A**) and accumulated at the SPs (**D**), while SEs of mutant lines (**B**,**C**,**E**,**F**) do not. They are visible in each CY-Ev Arabidopsis line (**G**–**L**, black arrows) as filamentous masses both in the SE lumen (**G**–**I**) and at the SPs (**J**–**L**) and numerous phytoplasmas are present in SEs (**G**–**I**) and at the SPs (**J**–**L**). *CC* companion cell, *PPC* phloem parenchyma cell, *phy* phytoplasmas, *P* plastids, *PPU* pore-plasmodesma unit, *SE* sieve element, *ser* sieve element reticulum, *SP* sieve plate. Bars correspond to 1 μm.

Twenty days after IAP, the H-Ev plants (Fig. 5A–F) contained SE protein filaments only in the wild-type individuals (Fig. 5A,D) and not in the mutants (Fig. 5B,C,E,F) as in the no-Ev plants (Fig. 3). By contrast, SEs of all CY-Ev lines were characterized by the presence of filaments (Fig. 5G–L). Phytoplasmas were abundant throughout the entire SE, both in the SE lumen (Fig. 5G–I) and in proximity of the sieve plates (Fig. 5J–L).

### Phytohormone quantification

To assess whether phytohormone-related defence mechanisms were activated in the Arabidopsis lines under investigation and to determine time and location of the activation, stress-related phytohormones were measured in extracts from no-Ev, H-Ev or CY-Ev plants, of wild-type, *Atseor1ko,* and *Atseor2ko* lines. For each condition, 6 plants were analysed and phytohormones were measured separately in laminae and midribs that were sampled at 5 days and 20 days after IAP (Tables 1, 2).

**Table 1:**
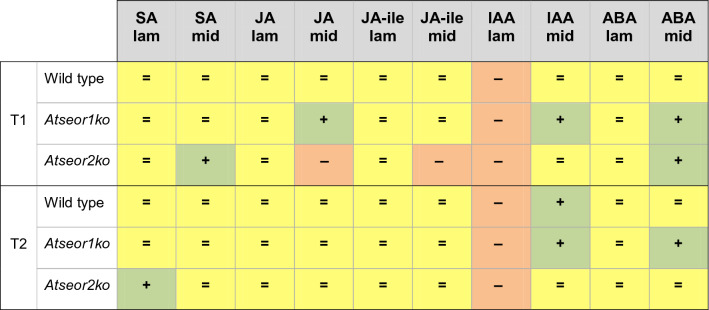
The table sums up the variations of phytohormone levels in the CY-Ev *Arabidopsis* lines during the infection compared to their respective H-Ev control plants.

T1 and T2 indicate, respectively, 5 and 20 days after the end of IAP.

= unchanged hormone level; + increased hormone level; − decreased hormone level.

**Table 2:**
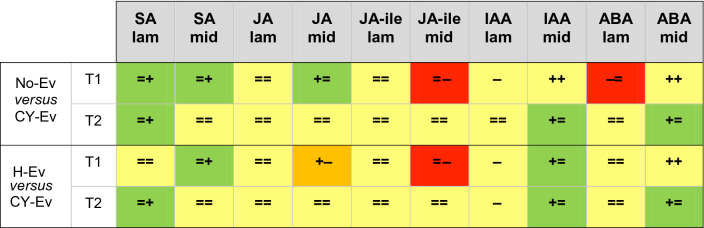
Responsiveness of *Atseor1ko* and *Atseor2ko* lines to phytoplasma infection (No-Ev, H-Ev or CY-Ev) at 5 (T1) and 20 (T2) days after the end of IAP.

== (yellow): no significant differences in both *Atseor1ko* and *Atseor2ko*.

++ (yellow): significant increase in both *Atseor1ko* and *Atseor2ko*.

−− (yellow): significant decrease in both *Atseor1ko* and *Atseor2ko*.

=+ (light green): no significant differences in *Atseor1ko*, significant increase in *Atseor2ko*.

= − (light red): no significant differences in *Atseor1ko*, significant decrease in *Atseor2ko*.

+= (light green): significant increase in *Atseor1ko*, no significant differences in *Atseor2ko*.

−= (light red): significant decrease in *Atseor1ko*, no significant differences in *Atseor2ko*.

+− (orange): significant increase in *Atseor1ko*, significant decrease in *Atseor2ko*.

**Figure 6:**
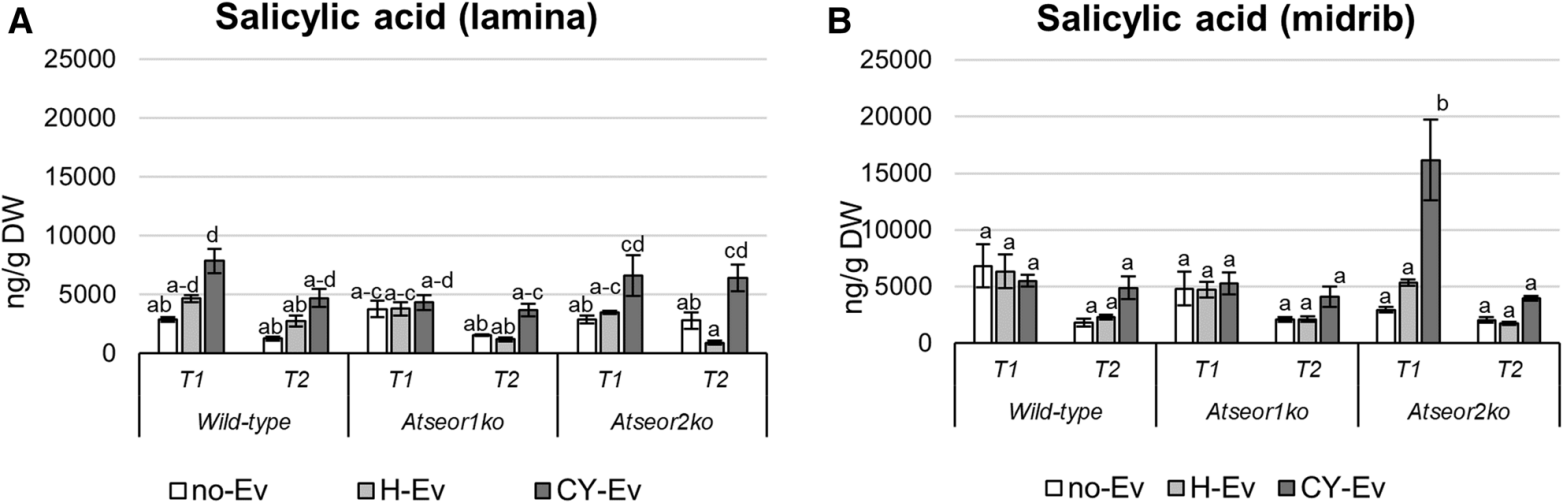
Salicylic acid levels in laminae and midribs of the three Arabidopsis lines non-infested by leafhoppers (no-Ev) or infested by healthy leafhoppers (H-Ev) or infested by CY-infected leafhoppers (CY-Ev), at the early (5 days after IAP, T1) and late (20 days after IAP, T2) stages of infection. The phytohormone levels are expressed as ng per g of tissue dry weight. Statistical analysis was performed using the Tukey HSD test as the post hoc test in a three-way ANOVA. Different letters (a, b, c) above the bars indicate significant differences, with *P* < 0.05; (a–c = abc, a–d = abcd). Error bars indicate the Standard Error of the Mean of 6 biological replicates for each condition.

HPLC–MS/MS analysis revealed that the SA level was not affected by *E. variegatus* infestation at both time intervals in both tissues of H-Ev plants (Fig. 6A,B). SA levels in the laminar tissues of all CY-infected lines did not change significantly (Fig. 6A), with exception of *Atseor2ko* plants at the late stage of infection. In midribs, a significant increase in the SA levels only occurred in *Atseor2ko* CY-EV plants at the early stage of infection (Fig. 6B).

**Figure 7:**
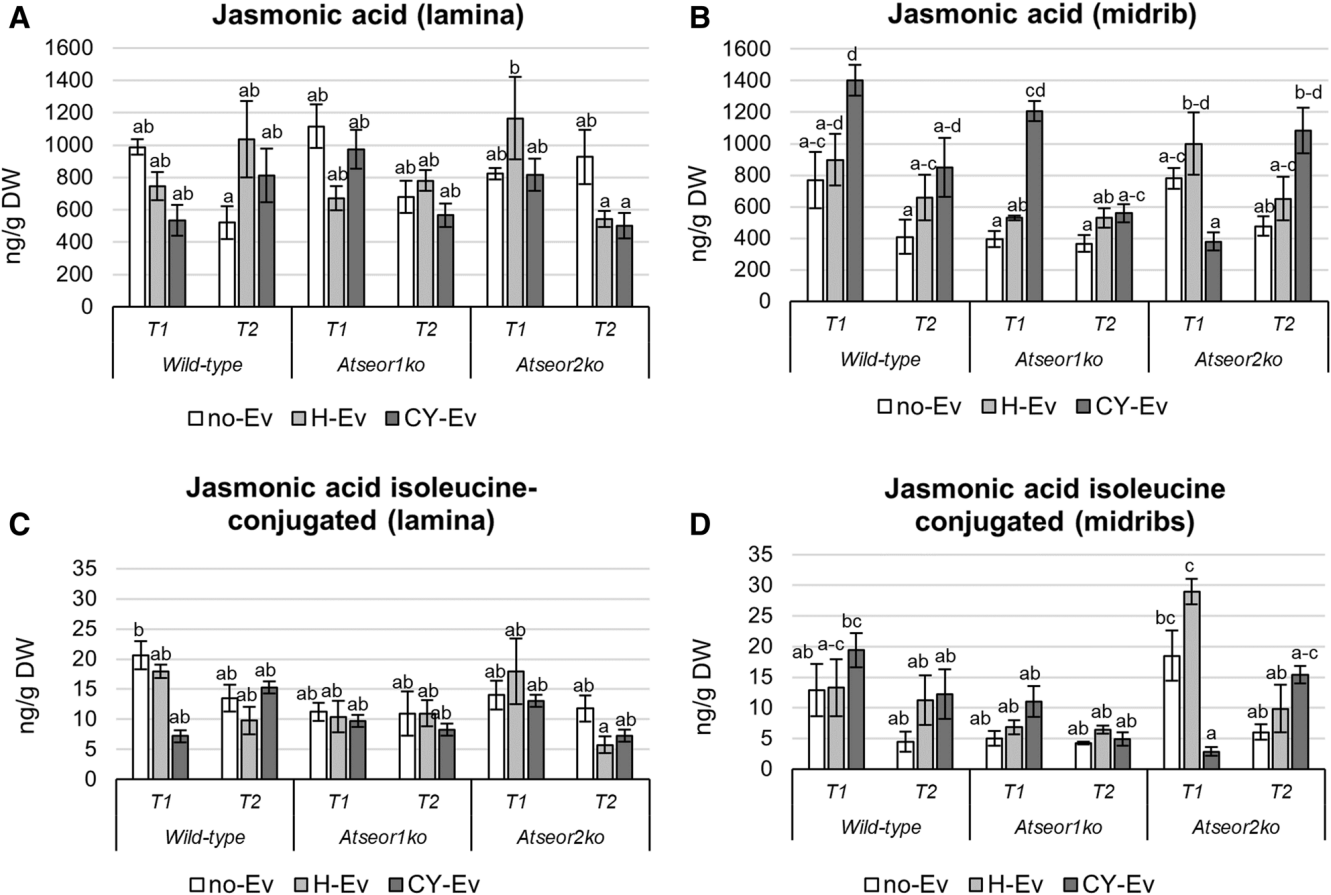
Jasmonic acid and jasmonate-isoleucine conjugate levels in laminae and midribs of the three Arabidopsis lines non-infested by leafhoppers (no-Ev) or infested by healthy leafhoppers (H-Ev) or infested by CY-infected leafhoppers (CY-Ev), at the early (5 days after IAP, T1) and late (20 days after IAP, T2) stages of infection. The phytohormone levels are expressed as ng per g of tissue dry weight. Statistical analysis was performed using the Tukey HSD test as the post hoc test in a three-way ANOVA. Different letters (a, b, c) above the bars indicate significant differences, with *P* < 0.05; (a–c = abc, a–d = abcd, b–d = bcd, c–e = cde). Error bars indicate the Standard Error of the Mean of 6 biological replicates for each condition.

The JA and JA-Ile levels were not affected by *E. variegatus* infestation at both time intervals in laminae and midribs of H-Ev plants (Fig. 7A–D). This insensitivity contrasted the significant increase in JA levels in CY-Ev midribs of *Atseor1ko* line as compared to those of H-Ev and no-Ev plants at the early stage of infection (Fig. 7B). *Atseor2ko* line showed an opposite trend: both JA and JA-Ile levels decreased at the early stage of infection in midribs. In laminae of all CY-infected lines JA and JA-Ile levels did not change in comparison with those of their controls (Fig. 7A,C).

**Figure 8:**
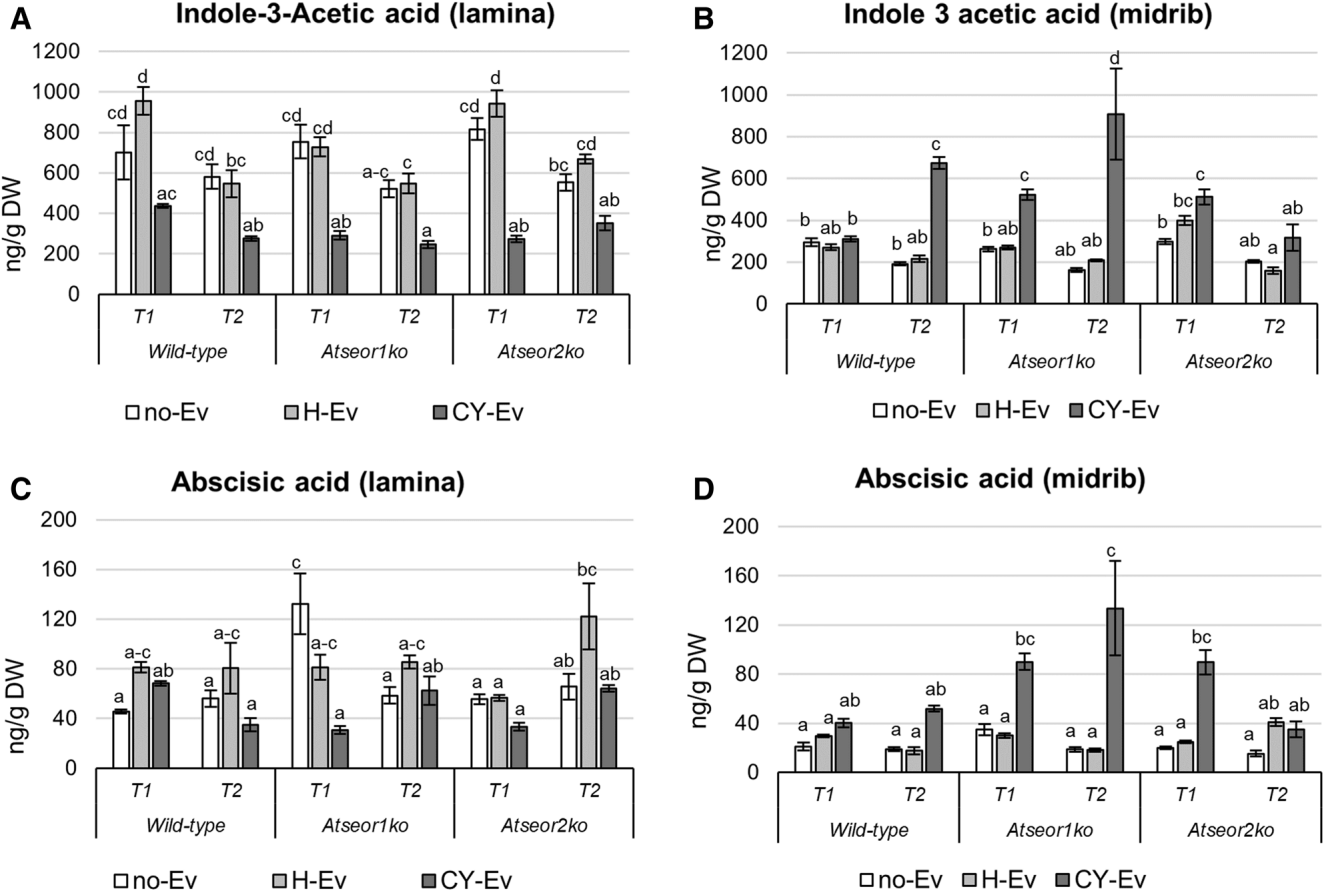
Indole acetic acid and abscisic acid levels in laminae and midribs of the three Arabidopsis lines non-infested by leafhoppers (no-Ev) or infested by healthy leafhoppers (H-Ev) or infested by CY-infected leafhoppers (CY-Ev), at the early (5 days after IAP, T1) and late (20 days after IAP, T2) stages of infection. The phytohormone levels are expressed as ng per g of tissue dry weight. Statistical analysis was performed using the Tukey HSD test as the post hoc test in a three-way ANOVA. Different letters (a, b, c, d) above the bars indicate significant differences, with *P* < 0.05; (a–c = abc). Error bars indicate the Standard Error of the Mean of 6 biological replicates for each condition.

The IAA levels were not affected by *E. variegatus* infestation at both time intervals in both tissues of H-Ev plants (Fig. 8A,B). The IAA concentrations in laminae decreased in the three CY-Ev lines 5 and 20 days after IAP (Fig. 8A), with a significant decrease in the two mutant lines at the early stage of infection. In midribs, the IAA concentration showed a statistically significant increase in wild-type CY-Ev samples, as compared to the H-Ev or no-Ev plants, 20 days after IAP (+ 190% was consistently enhanced). Interestingly, IAA level showed a significant increase at both time-points in the midribs of *Atseor1ko* plants (+ 93% at the early stage of infection and + 357% at the late stage).

The ABA levels were not affected by *E. variegatus* infestation at both time intervals in both tissues of H-Ev plants (Fig. 8C,D). Following phytoplasma infection (CY-Ev plants), no significant changes were found in laminae of Arabidopsis lines at both time points (Fig. 8C). In the midribs, ABA levels remained unchanged in wild-type plants at both time points and in *Atseor2ko* at late stage of infection (Fig. 8D).

In comparison with the No-Ev or H-Ev samples, the ABA concentrations were significantly increased in CY-Ev in the midribs at both time-points in *Atseor1ko* plants (+ 177% at the early infection stage and + 530% at the late infection stage) and at the early stage of infection in *Atseor2ko* plants (+ 242%).

All in all, infestation by *E. variegatus* does not seem to seriously affect the hormonal balance (Figs. 6, 7, 8), whereas CY infection is associated with changes on JA, IAA and ABA levels, in particular in the midribs of *Atseor1ko* plants during the pre-symptomatic early stage (Tables 1, 2).

## Discussion

### The *Atseor1ko* line limits phytoplasma replication from an early stage of infection onwards

In search for a role for SE protein filaments in response to phytoplasma infection^9^, phytoplasma-infected *Atseor1ko* plants turned out to host a significantly lower phytoplasma titre in comparison to wild-type and *Atseor2ko* lines, but a satisfactory explanation was not evident. AtSEOR1 and AtSEOR2 are thought to be necessary for filament formation through their heterodimeric interaction^8^. Therefore, it was speculated that in the *Atseor1ko* line, the AtSEOR2 protein in its free form, i.e. not linked to AtSEOR1, is involved in immune signalling through an interaction with defence-related plant proteins^10,11^. In line with this conjecture, AtSEOR2 was found to interact with AtRIN4, a PRR plasma membrane-anchored protein in a matrix-based yeast two-hybrid assay^10^. Expression of *AtRIN4*, and the associated *AtRPM1* and *AtRPS2* genes in healthy and phytoplasma-infected wild-type and *Atseor1ko* lines revealed an upregulation in the mutant line as compared to the wild-type, which was suggestive of a role of AtSEOR2 in promoting defence mechanisms^11^. In this frame, interactions between AtSEOR2 and diverse transcription factors^29^ as well as its intervention in the IAA and ABA signalling cascades were predicted^12,30^.

In the present work, the relation between AtSEOR2 and phytohormone synthesis and the impact on phytoplasma titres was investigated in wild-type, *Atseor1ko* and *Atseor2ko* Arabidopsis lines. As the effectiveness of the defence processes is related to the readiness of plants to counter pathogens^31^ and very little is known about the early response to phytoplasma infection, analyses were performed both at an early and a late time point of infection, i.e., respectively, at 5 days and 20 days after IAP. Up to 5 days after the end of IAP, the plant lines did not show any phenotypic differences (Fig. 1). As reported before^32^, growth and development of infected plants at this stage were comparable to those of control plants exposed to non-infected insect vectors.

Real-time PCR analyses evidenced that at the early stage (Fig. 2), the average phytoplasma titres were low (Cq values ± SE wild-type: 29.11 ± 2.17; *Atseor1ko*: n.d.; *Atseor2ko*: 30.98 ± 3.71), which has been demonstrated previously^33^. Interestingly, none of the *Atseor1ko* individuals tested positive at all for the presence of phytoplasma at this stage (Cq value: n.d. and Fig. 2A). The very high transmission efficiency of *E. variegatus* under natural and experimental conditions^34–37^ is confirmed in our experimental system (transmission rate 100%, p = 1^27^). Moreover, the higher vector survival rates on mutant lines (Fig. S1) together with the assumption that the vector fitness reflects the feeding capacity, infers that the lower phytoplasma titre in the *Atseor1ko* line is logically due to plant properties and not to reduced insect-mediated transmission efficiency.

The fact that insects showed higher survival rate on infected mutant lines than in wild-type, could be due to the complementary capability of AtSEOR proteins (both expressed in wild-type Arabidopsis) to aggregate in presence of phytoplasmas [this work;^9^], reducing the phloem mass-flow and impairing stylet sucking-activity. Will et al.^38^ demonstrated that SEOR-mediated plugging is induced by green peach aphid (Myzus persicae) feeding on Vicia faba and this mechanism impairs feeding in aphid-resistant varieties^39^.

Phytoplasma titres at the early stage of infection showed trends similar to those 20 days after IAP (when the symptoms had become manifest): the phytoplasma titres in wild-type or *Atseor2ko* plants exceeded by far the titre in *Atseor1ko* plants (Fig. 2 and^9^). Therefore, phytoplasma multiplication was presumably impaired in the *Atseor1ko* line from the earliest stages of infection onwards.

### Sieve-element protein filaments aggregate in sieve tubes of wild-type Arabidopsis line from the early stage of infection onward

We previously demonstrated that SE protein filaments play a role in the plant response to phytoplasma infection, even in the absence of genes that are considered necessary for their formation in healthy plants^8,9^. Nevertheless, the picture drawn at that time did not explain whether SE protein filaments per se are involved in some defence mechanisms, such as early pathogen containment. Ultrastructural analysis confirmed the presence, at the late stage of infection (i.e., 20 days after IAP, Fig. 5), of filamentous structures in each CY-Ev Arabidopsis line (wild-type, *Atseor1ko* and *Atseor2ko*) and revealed that, at the first stage of infection (5 days after IAP), SE protein filaments only aggregated in CY-Ev wild-type plants (Fig. 4). Their initial absence in the Arabidopsis line that is best equipped to suppress the pathogen invasion from the early stage of infection on (i.e.* Atseor1ko*), led us to exclude aggregation of SE protein filaments as a possible explanation for the better defence performance of the *Atseor1ko* line. Therefore, our investigations were further focused on the phytohormone levels, which are frequently related to defence mechanisms^15,16,40–43^. Since leaf midribs are rich in phloem tissues, where phytoplasmas and plants physically and chemically interact^5^, plant responses were determined in leaf midribs and laminae separately.

### Phytohormone levels in Arabidopsis lines are not affected by *E. variegatus* infestation at 5 and 20 days after the end of the IAP

Damage inflicted by insect feeding leads to the immediate activation of phytohormone-related signaling^26^ and in particular to the synthesis and accumulation of JA in tissues both proximal and distal to injury sites^44^.

As for the effects of leafhopper infestation, phytohormone levels were equal in the midribs or laminae of each line and at both time points (i.e. 5 and 20 days after the end of IAP) in H-Ev and no-Ev samples. Changes in phytohormonal balance following leafhopper infestation have been amply described^26^, but the results were quite variable, probably owing to variations in infestation times^45^. In general, defense phytohormones are immediately induced in plant tissues after recognition of invaders, but the amounts tend to level off during persistent insect infestation^44^. Moreover, the effects of cutting, necessary to isolate midribs from laminae, may overshadow insect-induced responses^44^.

The fact that the phytohormone levels are identical in H-Ev and no-Ev plants strongly supports the conclusion that the phytohormone modulation in CY-Ev plants is solely due to phytoplasma infection. This is in agreement with the observation that 6 days after *Scaphoideus titanus* infestation in grapevine, the expression level of genes involved in JA and ABA pathways were similar to those found in non-infested control leaves, while they had been upregulated 3 days after infestation^46^.

### Jasmonates, but not salicylic acid seem to be involved in the response of *Atseor1ko* line to phytoplasma infection

There are several indications for phytohormone involvement in diverse phytoplasma-plant interactions^4^, but few studies have addressed plant responses at the early stage of infection^40,46^.

The most frequently studied phytohormones in relation with phytoplasma infections are SA and JA, which both confer signal transduction leading to plant resistance. JA- and SA-mediated signalling pathways are presumed to be antagonistic^41,42^. Traditionally, SA signalling is deemed to activate resistance against biotrophic and hemibiotrophic pathogens, while JA is mainly thought to induce resistance against necrotrophic pathogens and wounding^44,47^.

In CY-Ev wild-type and *Atseor1ko* lines SA levels did not change significantly in all tissues examined (Fig. 6). SA increased in the midribs of *Atseor2ko line* at the early stage of infection, followed by a drop at the late stage (Fig. 6). Enhanced amounts of SA resulted from different plant–phytoplasma interactions in whole leaves^18,40,48^, in midribs^22^ and phloem sap^49^. On the other hand, cases, which showed reduced SA levels in response to phytoplasma infection were also described^17,50^. The reason why SA level was significantly higher in *Atseor2ko* line compared to the other lines is unknown. It has to be noted that SA levels depend on many factors, such as the developmental stage of the vegetative cycle^17^, varying environmental conditions^20^, the phytoplasma strain in question^51^, and distinct sets of virulence factors^49^.

With regard to the JA concentration, the levels of both jasmonates were virtually unaffected in laminae. In midribs of all *Arabidopsis* lines JA showed a non-significant increment, apart from the *Atseor1ko* line, which showed significantly higher jasmonate levels at an early stage of infection (Fig. 7B). An increase of JA levels during the early stages of infection process, followed by decrease at symptom appearance, was described in different plant–phytoplasma interactions^14,17,20,24,52^.

Sugio et al.^53^ found that *Arabidopsis* plants infected with the ‘*Ca*. P. asteris’ strain AY-WB produced more JA in old asymptomatic leaves (as well as in uninfected plants) as compared to young symptomatic leaves. The authors also demonstrated that reduced JA levels affected plant development, leading to symptom appearance and increasing insect-vector fitness^53^. Decreased JA levels were also reported in Arabidopsis expressing phytoplasma virulence factors (i.e. TENGU^14^ or SAP11^53^), indicating that phytoplasma effectors were involved in host hormonal changes.

Janik et al.^17^ hypothesized a relationship between an increased phytoplasma titre, the activity of phytoplasma effectors and the decrease of JA levels over the growth season. Interestingly, in perennial plants, increased JA synthesis was correlated to the phenomenon of recovery^40,48^, a resilience status characterized by the loss of disease symptoms in plants which previously showed them^54,55^. Hence, the higher content of JA in *Atseor1ko* line could be related to failing phytoplasma detection (and to the absence of activity by their effectors) at the first stage of infection.

A JA–SA antagonism^41,42^ may be visible in *Atseor1ko* and *Atseor2ko* lines at the early stage of infection, because at the high JA levels in the one correspond the low SA content in the other and vice versa at the same time point (Figs. 6B, 7B).

### IAA- and ABA-synthesis are activated in the midribs of the phytoplasma-infected *Atseor1ko* line

As for the IAA variations in phytoplasma-infected plants, expression analyses of IAA-related genes in whole leaves^56,57^, buds^58^, or leaf midribs^59^ infer a downregulation in several infected hosts. On the other hand, IAA levels were reported to increase in phloem sap^49^, whole leaves^60^ and leaf midribs^59^ of phytoplasma-infected plants as compared to the respective control samples.

Here, phytoplasma infection induced a decrease in IAA levels in the laminae and an increase in the midribs in each *Arabidopsis* line (Fig. 8). This indicates a positive response of IAA synthesis, located in the midribs as reported previously^59^. The increase in IAA was statistically significant in infected midribs of *Atseor1ko* mutants at both time points, i.e. at 5 and 20 days after IAP (Fig. 8).

Previous studies evidenced an increase in ABA levels and the upregulation of ABA-related genes in all tissues of phytoplasma-infected plants^17,18,49,58,61^. In our study, a tissue-dependent ABA response was observed in wild-type plants; the ABA level decreased in laminae, while it increased in midribs. Variations in the expression of genes related to ABA biosynthesis have been associated with symptom expression^61^. ABA-promoted stomatal closure could induce pre-invasive defence by inhibiting the entry of pathogens through passive ports^62,63^. Furthermore, ABA signalling would initiate events such as callose accumulation and antagonize the signalling cascades of other phytohormones at an advanced state of infection^64,65^. It is worth noting that *Atseor1ko* was the only line, in which ABA is significantly enhanced in the midrib from the early infection stage on, and the higher level was maintained throughout the entire measurement period.

### Potential modes of involvement of AtSEOR2

Thus far, the relationship between free AtSEOR2 and phytohormone synthesis is a mystery. Yet, there is a wide range of possibilities.

According to the COACH platform^66^, AtSEOR2 protein (but not AtSEOR1) may have Ca^2+^ binding sites, which suggests a possible role for AtSEOR2 in the lowering of Ca^2+^ levels as does the sequestration of Ca^2+^ ions by SEOR-based forisomes in legumes^67^. The subsequent modification of Ca^2+^ signatures may promote ABA and IAA synthesis^68–72^ in *Atseor1ko* infected plants. This hypothesis seemingly makes a logical connection between AtSEOR2 and the low phytoplasma titre owing to enhanced IAA and ABA synthesis.

In addition or alternatively, AtSEOR2 may effect on the IAA and ABA signalling pathways. To the best of our knowledge, the number of reports about possible interaction of AtSEOR2 protein with components of IAA- or ABA-signalling cascade is scarce. Yeast two-hybridization experiments demonstrated that AtSEOR2 is able to interact with the At4g04950 gene product, a monothiol glutaredoxin that is a key component involved in ROS accumulation and IAA signalling^73^. The role of AtSEOR2 in ABA-cascade signalling has also hardly been explored thus far. Some evidence has recently been presented for an interaction between AtSEOR2 and SUA (SUPPRESSOR OF ABI3-5), a main component of the ABA signalling pathway^74^, and its involvement in the increased sensitivity to ABA^74^. Moreover, Nakashima and co-workers^30^ demonstrated that *AtSEOR2* is one of the many genes whose expression changes in *A. thaliana* knock-out mutants of three SnRK2 kinases involved in ABA signalling. Interestingly, AtSEOR2 has been reported to interact with the At1G31280.1 gene product^12^, an ABA-regulated protein that controls plant response against virus infections^75^.

Furthermore, it is not excluded that AtSEOR2 directly interacts with receptor(s) in the SE-CC complex to elicit defence responses. SEOR proteins are characterized by a conserved C-terminal M1 motif, containing several conserved cysteine residues^28^ characteristic of the so-called peptide ligands^76^, which are engaged in the regulation of developmental processes and defence mechanisms against pathogens^77^. Interestingly, in mulberry infected by yellow dwarf disease, a phytoplasma-responsive gene encoding a protein that shows structural similarity to peptide ligands, was identified. This gene is involved in signaling and metabolism of IAA, ABA and JA^78^.

In conclusion, AtSEOR2 indirectly manipulates plant response via increased phytohormone synthesis and phytohormone signalling and perhaps via interaction with membrane receptors. These responses emerge very early in the infection process, long before the appearance of infection symptoms.

## Methods

### Plant material and insect vectors

The seeds of wild-type, *Atseor1ko* (SALK_081968C), and *Atseor2ko* (SALK_148614C) lines of *A*. *thaliana* plants ecotype Columbia^9^ were obtained from the Nottingham Arabidopsis Stock Centre (NASC). Wild-type and mutant lines (72 plants of each line) were grown at 20/22 °C under short-day conditions (9 h L/15 h D). Before the experiment, plants were cultivated for 45 days on a 5:1 mixture of soil substrate and perlite and fertilized twice a month with an N–P–K liquid fertilizer.

Healthy colonies of the insect vector *E. variegatus* were reared on *Avena sativa* in vented plexiglass cages at 20/22 °C, under short-day conditions (9 h L/15 h D)*.* Fourth and fifth instar nymphs were transferred to *Chrysanthemum carinatum* plants infected with a phytoplasma strain related to ‘*Candidatus* Phytoplasma asteris’ (‘*Ca*. P. asteris’, 16SrI-B subgroup), called Chrysanthemum yellows (CY) phytoplasma^9^ as the source of inoculum for a 7-day phytoplasma acquisition-access period (AAP). After the AAP, the insects were fed again on *A. sativa* for the 35-day latency period (LP) after which they have become infectious. Twelve 45-day-old *A. thaliana* plants per line were then each exposed to three infectious insects (CY-infected *E. variegatus*, CY-Ev plants) for a 7-day phytoplasma inoculation-access period (IAP), after which the insects were manually removed. Twelve Arabidopsis plants per line, treated with three healthy leafhoppers (H-Ev plants) were used as a healthy control. Healthy leafhoppers have been collected from healthy colonies and were as old as the infected ones.

For microscopy and phytohormone analyses, 12 plants per line not subjected to insect feeding (non-infested plants, no-Ev plants), were included as additional negative controls. For ultrastructural observations and phytohormone quantification, 6 H-Ev and 6 CY-Ev plants from each line (i.e. 6 independent biological replicates) were used, for the phytoplasma titre analyses12 H-Ev and 12 CY-Ev plants (i.e. 12 independent biological replicates) were used.

### Evaluation of insect survival rate and phytoplasma detection

To ascertain successful phytoplasma transmission to the Arabidopsis plants, survival rates and the presence of phytoplasma were checked in leafhoppers that were removed from Arabidopsis plants at the end of the IAP. The survival rate was calculated using 12 biological replicates (i.e. 12 *Arabidopsis* plants) for each condition (i.e. various Arabidopsis lines and infection times). For phytoplasma detection, the 3 insects used on each Arabidopsis were pooled, to obtain 72 pools per condition. Total DNA was extracted as described^79^ and the presence of phytoplasmas in each pool was assayed by conventional PCR using the primer pair R16F2/R2, as described by Pagliari and co-authors^9^.

Phytoplasma transmission by leafhoppers was evaluated on the basis of the number of Arabidopsis plants showing symptoms 20 days after the end of IAP. To estimate the proportion of infectious insects in our experiment, the maximum likelihood estimator of p, p = 1 − Q^1/k^ was used^27^, where Q is the observed fraction of non-infected plants and k is the number of insects per plant, assuming that the vectors acted independently^27^. The formula can be applied when transmission trials are carried out using groups of insects^35^.

Statistical analysis was performed using SigmaPlot 12.0 software (Systat Software, Inc., San Jose, CA, USA). The normal distribution of the data was checked with the Shapiro–Wilk normality test. A three-way ANOVA of the means (from 12 biological replicates and 3 technical replicates) followed by the Holm-Sidak test as the post hoc test for multiple comparisons demonstrated the significance for *p* < 0.05.

### Phytoplasma quantification in Arabidopsis

Total DNA was extracted from 200 mg of whole-leaf tissue of H-Ev and CY-Ev plants according to Martini et al.^80^. The amount of CY phytoplasmas was quantified according to a real-time PCR protocol described in detail by Pagliari and co-authors^9^. Briefly, the ribosomal protein gene *rplV* (*rpl22*) was the target for amplification of CY phytoplasma DNA using the primer pair rp(I-B)F2/rp(I-B)R2^9^) and a CFX96 real-time PCR detection system (Bio-Rad Laboratories, Richmond, CA, USA). A standard curve was established by tenfold serial dilutions of plasmid DNA containing the 1,260 bp ribosomal protein fragment from CY phytoplasma, amplified with the primer pair rpF1C/rp(I)R1A. Real-time PCR mixture and cycling conditions were as previously described^9^. The phytoplasma concentration was expressed as the number of CY phytoplasma genome units (GUs) per mg of leaf sample to normalize the data. Differences among the means were calculated using SigmaPlot 12.0 software (Systat Software). The normal distribution of the data was checked with the Shapiro–Wilk normality test. A two-way ANOVA of the means (obtained from 12 biological replicates and 3 technical replicates) followed by the Holm-Sidak test as post hoc test for multiple comparisons demonstrated the significance for *p* < 0.05.

### Transmission electron microscopy

To preserve the damage-sensitive sieve-element ultrastructure, a gentle preparation method was adopted following Pagliari et al.^9^. From each plant a 25 mm-long midrib portion was excised from rosette leaves. The midrib segments were submerged in MES buffer and then fixed with 3% paraformaldehyde and 4% glutaraldehyde solutions. Samples were rinsed, post-fixed overnight with 2% (w/v) OsO4, dehydrated in a graded ethanol series and then transferred to propylene oxide. From the central part of each midrib, 6–7 mm long segments were excised and embedded in Epon/Araldite epoxy resin (Electron Microscopy Sciences, Fort Washington, PA, USA).

Ultrathin sections (60–70 nm in thickness) were cut, stained with UAR-EMS (uranyl acetate replacement stain) (Electron Microscopy Sciences), and observed under a PHILIPS CM 10 (FEI, Eindhoven, The Netherlands) transmission electron microscope (TEM), operated at 80 kV, and equipped with a Megaview G3 CCD camera (EMSIS GmbH, Münster, Germany). Five non-serial cross-sections from each sample were analysed.

### Phytohormone analyses

We adopted a validated HPLC–MS/MS method^81^, optimized for *A. thaliana* and the low concentrations (nM to μM) of the phytohormones of interest. Phytohormone extraction was performed using 6 plants per experimental condition.

For each sample, roughly 250 mg of midribs and 250 mg of laminae were collected for phytohormone analysis, immersed immediately in liquid nitrogen and then stored at − 80 °C, as described by Pommerrenig et al.^82^.

After freeze-drying, the samples were homogenized in a Geno/Grinder 2010 (SPEXSample Prep, München, Germany) at 1,100 rpm for 60 s. After homogenization, the phytohormones salicylic acid (SA), abscisic acid (ABA), jasmonic acid (JA), jasmonic acid- isoleucine conjugate (JA-Ile) and indole acetic acid (IAA) were extracted from 10–20 mg of dried plant tissue using 1 mL of extraction solution containing 20 ng/mL d6-ABA, 10 ng/mL d5-IAA, 20 ng/mL d6-JA and 10 ng/mL d4-SA as internal standards. After mixing and centrifugation, the supernatants were evaporated in a Speed Vac at 45 °C and the pellets resuspended in 100 μL of methanol:water 1:1.

The extracts were analysed using an HPLC–MS/MS method^81^ on an Agilent 1,100 HPLC system (Agilent Technologies, Böblingen, Germany) connected to a LTQ Ion Trap mass spectrometer (Thermo Scientific, Bremen, Germany). Chromatographic separation was carried out in a Luna phenyl-hexyl column (150 × 4.6 mm, 5 μm; Phenomenex, Aschaffenburg, Germany). Formic acid (0.05%, v/v) and MeOH with 0.05% (v/v) formic acid were used as mobile phases A and B, respectively. The elution profile was: 0–10 min, 42–55% B in A; 10–13 min, 55–100% B; 13–15 min 100% B; 15–15.1 min 100–42% B in A; 15.1–20 min 42% B in A. The mobile phase flow rate was 1.1 mL/min. The injection volume was 20 μL.

Phytohormone quantifications were based on calibration curves, and the data obtained from each sample were subsequently analysed with XCalibur software (Thermo Fisher Scientific). Statistical differences between the means obtained from 6 individuals exposed to the different conditions (i.e. various Arabidopsis lines and infection times) were evaluated using R Studio 1.1.456 software (Northern Ave, Boston, MA, USA) using three-way ANOVA and the Tukey HSD test as post hoc test for pairwise multiple comparisons, with *p* < 0.05. The normal distribution of the data was checked with the Shapiro–Wilk normality test.

## Supplementary information

Supplementary Information.
