# Supplementary material for: Pre-symptomatic modified phytohormone profile is associated with lower phytoplasma titres in an Arabidopsis seor1ko line
Source: Sci Rep. 2020 Sep 8;10:14770. doi: 10.1038/s41598-020-71660-0 (PMC7479616; doi:10.1038/s41598-020-71660-0)
Supplement: Supplementary file 1 — Supplementary Information. [file 41598_2020_71660_MOESM1_ESM.docx]

Pre-symptomatic modified phytohormone profile is associated with lower phytoplasma titres in an Arabidopsis *seor1ko* line

Chiara Bernardini^1^, Laura Pagliari^1^, Valeria De Rosa^1^, Marilia Almeida-Trapp^2^, Simonetta Santi^1^, Marta Martini^1^, Sara Buoso^1^, Alberto Loschi^1^, Nazia Loi^1^, Fiorella Chiesa^1^, Axel Mithöfer^3^, Aart J. E. van Bel^4^, Rita Musetti^1^*

^1^Department of Agricultural, Food, Environmental and Animal Sciences, University of Udine, via delle Scienze, 206 I-33100 Udine, Italy

^2^Department of Bioorganic Chemistry, Max Planck Institute for Chemical Ecology, Hans-Knöll-Straße 8, D-07745 Jena, Germany

^3^Research Group Plant Defense Physiology, Max Planck Institute for Chemical Ecology, Hans-Knöll-Straße 8, D-07745 Jena, Germany

^4^Institute of Phytopathology, Justus-Liebig University, Heinrich-Buff-Ring 26–32, D-35392 Giessen, Germany

*** Correspondence:** Prof. Rita Musetti rita.musetti@uniud.it

**
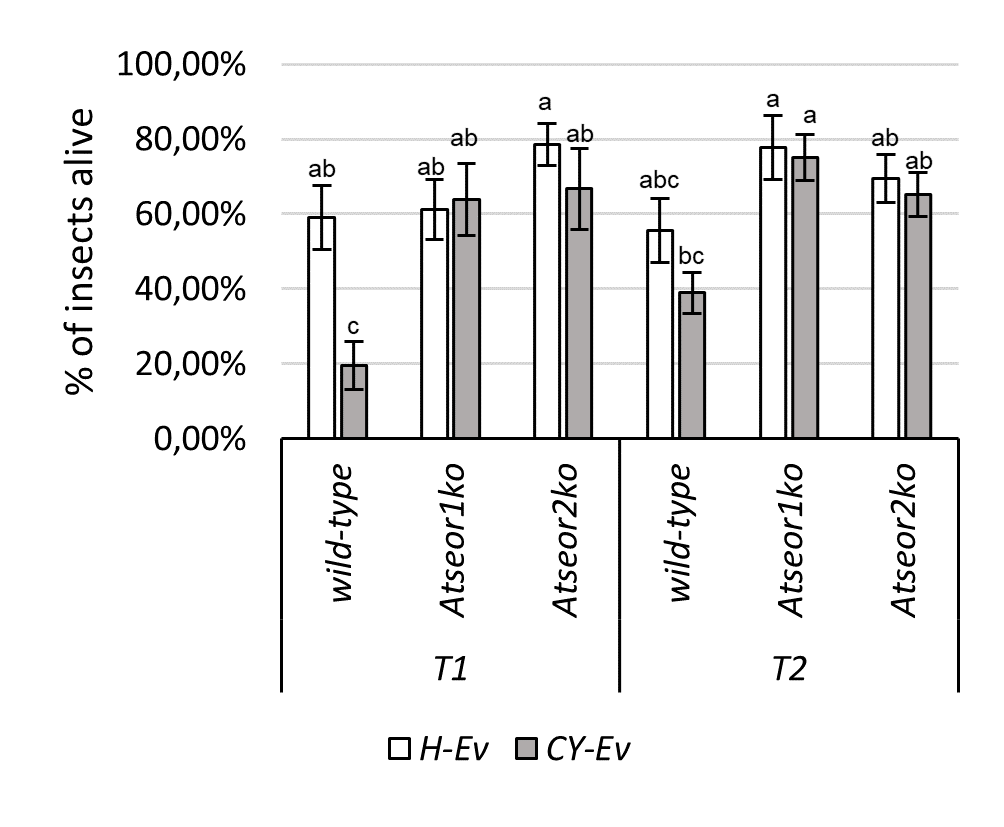
**

**Fig. S1. *Euscelidius variegatus* survival rates on three Arabidopsis lines at the early (5 days after IAP, T1) and late (20 days after IAP, T2) stage of infection.** The leafhopper survival rate is expressed as the percentage of living insects against the number of insects *per* plant line. Healthy leafhoppers (H-Ev) were used as controls for the survival of CY-infected leafhoppers (CY-Ev). Different letters (A; B; C) above the bars indicate significant differences according to Dunn’s test, P<0.05. Error bars indicate Standard Error of the Mean of 12 biological replicates for each condition.
